# Complete Genome Sequences of Infectious Bronchitis Virus Genotype JP-II (GI-7) and JP-III (GI-19) Strains Isolated in Japan

**DOI:** 10.1128/mra.00670-22

**Published:** 2023-01-04

**Authors:** Masaji Mase, Kanae Hiramatsu, Satoko Watanabe, Hiroshi Iseki

**Affiliations:** a National Institute of Animal Health, National Agriculture and Food Research Organization, Tsukuba, Ibaraki, Japan; b United Graduate School of Veterinary Sciences, Gifu University, Gifu, Gifu, Japan; c Graduate School of Life and Environmental Sciences, Osaka Prefecture University, Izumisano, Osaka, Japan; d Oita Livestock Hygiene Service Center of Oita Prefecture, Oita, Oita, Japan; Katholieke Universiteit Leuven

## Abstract

We report the complete genome sequences of strains JP/Yamanashi/93 and JP/Shimane/98, which are classified in JP-II (GI-7) and JP-III (GI-19), respectively, the major genotypes of infectious bronchitis virus (IBV) in Japan. This information will be useful for the in-depth understanding of the evolution of IBV in Japan.

## ANNOUNCEMENT

Avian infectious bronchitis virus (IBV) causes a highly contagious respiratory and urogenital disease that affects egg production and shell quality in layer chickens. IBV belongs to the genus *Gammacoronavirus* in the family *Coronaviridae* of the order *Nidovirales* (1).

The genetic grouping of IBV has been performed based on the nucleotide sequence of the S1 region of the spike glycoprotein gene (2–4). This glycoprotein mediates cell attachment and is a major target of neutralizing antibodies in chickens (5, 6). A classification scheme based on analysis of the S1 gene has been proposed, including grouping and naming 32 lineages, comprising six genetic groups (4). In Japan, seven Japanese genotypes, namely, JP-I, JP-II, JP-III, JP-IV, Mass, Gray, and 4/91, have been reported (7, 8). These genotypes were confirmed to correspond to lineages GI-18, GI-7, GI-19, GVI-1, GI-1, GI-3, and GI-13, respectively, based on the aforementioned classification scheme (9).

In addition to genotypes JP-I, Mass, and Gray, genotypes JP-II and JP-III have been confirmed since the 1980s and 1990s, respectively (7). In Japan, however, to date the complete genome has been determined only for genotype JP-I (10, 11), and only partial sequences are available for genotypes JP-II and JP-III. Here, the complete nucleotide sequences of the major genotype JP-II and JP-III strains, which were isolated from chickens, were determined.

In 1993, the JP/Yamanashi/93 strain was isolated from the kidney of a dead chicken in Yamanashi prefecture in central Japan; this was classified as JP-II genotype as described previously (7). In 1998, the JP/Shimane/98 strain was isolated from the trachea of a dead chicken with respiratory signs in Shimane prefecture in western Japan; this was classified as JP-III genotype as described previously (7).

In this study, these viruses were grown in embryonated chicken eggs and used for genetic analysis at the fourth passage. Viral RNA was extracted from the infected fluids using a QIAamp viral RNA minikit (Qiagen), and cDNA was synthesized using random hexamer primers. PCR was performed to amplify the cDNA with *Ex Taq* DNA polymerase (TaKaRa, Tokyo, Japan). As described in previous studies, specific primers were used for genome sequencing with the Sanger method (10–13). Thirty-one pairs of primers were used. The amplicons were sequenced in both directions. Both the 5′ and 3′ termini of the genome were determined using a rapid amplification of cDNA ends (RACE) kit (Invitrogen).

The sequenced fragments were quality checked and assembled using ATGC-Mac v5 (Genetyx Corp., Japan). The lengths of the complete genomes of strains JP/Yamanashi/93 (JP-II) and JP/Shimane/98 (JP-III), excluding the poly(A) tail, were 27,605 nucleotides (nt), with a G+C content of 38.29%, and 27,643 nt, with a G+C content of 38.03%, respectively. The genomes of both strains have the typical genetic structure of the reported IBV strains (14) (Table 1).

**TABLE 1:** Numbers of nucleotides in each open reading frame of representative IBV genotype strains

ORF^*a*^	No. of nucleotides in ORF in strain:							
	JP/Yamanashi/93 (genotype JP-II [GI-7] [GenBank accession no. LC716900])	JP/Shimane/98 (genotype JP-III [GI-19] [GenBank accession no. LC716901])	JP/KH/64 (genotype JP-I [GI-18] [GenBank accession no. LC634083])	H120 (genotype Mass [GI-1] [GenBank accession no. MN548287])	Gray/60 (genotype Gray [GI-3] [GenBank accession no. GU393334])	QX (genotype QX [GI-19] [GenBank accession no. MN548289])	CR88 (genotype 4/91 [GI-13] [GenBank accession no. MN548285])	ck/CH/IBTZ/2012 (genotype GVI-I [GenBank accession no. KF663559])
1a	11,859	11,862	11,859	11,802	11,859	11,850	11,859	11,862
1ab	19,893	19,896	19,893	19,836	19,893	19,884	19,893	19,896
S	3,492	3,495	3,510	3,489	3,504	3,498	3,468	3,516
3a	174	174	174	174	174	174	174	174
3b	192	195	195	195	195	192	195	189
3c	279	330	324	330	324	333	285	312
M	678	681	672	678	666	777	678	681
4b	285	285	285	243	294	285	285	285
4c	171	162	171	171	171	147	171	147
5a	198	198	198	198	198	198	201	198
5b	249	249	249	249	249	249	249	249
N	1,230	1,230	1,230	1,230	1,233	1,230	1,230	1,230
6b	153	219	222	231	ND^*b*^	222	225	222

a ORF, open reading frame.

b ND, not detected.

A BLAST search of the complete genome of the JP/Yamanashi/93 strain showed 96.01% similarity to that of the JP/Toyama/2000 strain (11); on the other hand, that of the JP/Shimane/98 strain showed 94.59% similarity to that of the JP/Yamanashi/93 strain. This information will be useful for the in-depth understanding of the evolution of IBV in Japan.

### Data availability.

The genome sequences were deposited in GenBank under the accession numbers LC716900 and LC716901.
